# Correction: Hwang et al. *IFN-β* Overexpressing Adipose-Derived Mesenchymal Stem Cells Mitigate Alcohol-Induced Liver Damage and Gut Permeability. *Int. J. Mol. Sci.* 2024, *25*, 8509

**DOI:** 10.3390/ijms27188423

**Published:** 2026-09-21

**Authors:** Soonjae Hwang, Young Woo Eom, Seong Hee Kang, Soon Koo Baik, Moon Young Kim

**Affiliations:** 1 Department of Biochemistry, Lee Gil Ya Cancer and Diabetes Institute, College of Medicine, Gachon University, Incheon 21999, Republic of Korea; soonjae@gachon.ac.kr; 2 Regeneration Medicine Research Center, Wonju College of Medicine, Yonsei University, Wonju 26426, Gangwon-do, Republic of Korea; yweom@yonsei.ac.kr (Y.W.E.); baiksk@yonsei.ac.kr (S.K.B.); 3 Cell Therapy and Tissue Engineering Center, Wonju College of Medicine, Yonsei University, Wonju 26426, Gangwon-do, Republic of Korea; 4 Department of Internal Medicine, College of Medicine, Korea University, Seoul 02841, Republic of Korea; shkang0114@gmail.com; 5 Department of Internal Medicine, Wonju College of Medicine, Yonsei University, Wonju 26426, Gangwon-do, Republic of Korea

The journal’s Editorial Office and Editorial Board are jointly issuing a resolution and a removal of the Journal Notice linked to this article [1]. Following concerns raised about the integrity of the peer review (misconduct by one of two reviewers), the Editorial Office has conducted a post-publication peer review of this article. This process included the recruitment of a new independent reviewer and was supervised by the original Academic Editor to ensure full compliance with MDPI’s Editorial Process (https://www.mdpi.com/editorial_process (accessed on 6 September 2026)). The authors were unaware of misconduct by the reviewer and had not included any suggestions by the reviewer for inappropriate references.

As a result of this process, the Academic Editor and the authors have agreed to update the following aspects of this publication:

Reviewer report 2 has been removed from the peer-review record, while one new review report has been added and uploaded to the peer-review record (https://www.mdpi.com/1422-0067/25/15/8509/review_report).

Based on the new review report, the following corrections have been made to the original publication:

## Addition of References

In the original publication [1], two references were not included. The following references have been added to the reference list:

30. Jang, Y.O.; Kim, Y.J.; Baik, S.K.; Kim, M.Y.; Eom, Y.W.; Cho, M.Y.; Park, H.J.; Park, S.Y.; Kim, B.R.; Kim, J.W.; et al. Histological improvement following administration of autologous bone marrow-derived mesenchymal stem cells for alcoholic cirrhosis: A pilot study. *Liver Int.* **2014**, *34*, 33–41.
31. Suk, K.T.; Yoon, J.H.; Kim, M.Y.; Kim, C.W.; Kim, J.K.; Park, H.; Hwang, S.G.; Kim, D.J.; Lee, B.S.; Lee, S.H.; et al. Transplantation with autologous bone marrow-derived mesenchymal stem cells for alcoholic cirrhosis: Phase 2 trial. *Hepatology* **2016**, *64*, 2185–2197.

Consequently, references [30–42] in the original publication have been renumbered as [32–44]. The in-text citations have been updated accordingly.

## Text Correction

In the original publication [1], a paragraph setting out the specific knowledge gaps addressed by this study was missing from Section 1 (Introduction). A new paragraph has been added before the final paragraph of the Introduction and reads as follows:

“Two questions therefore remain unanswered. First, it is unclear whether engineering ASCs to overexpress IFN-β offers any advantage over conventional, unmodified ASCs in ALD. Clinical trials of mesenchymal stem cells (MSCs) in alcoholic cirrhosis have produced histological benefit that is real but partial and variable [30,31], suggesting that the paracrine output of unmodified cells may be insufficient. Because IFN-β acts upstream of the STAT1 axis, its overexpression is expected to amplify the secretion of HGF—a barrier-repairing factor—together with TRAIL, rather than a single downstream effector, and thereby to act on the gut–liver axis rather than on the liver alone. Second, it is unclear whether such an enhancement can be achieved without a viral vector. Non-viral delivery avoids the insertional mutagenesis and vector-directed immune responses that currently limit the clinical translation of virally engineered ASCs. Addressing these two gaps is the rationale for the present study.”

In addition, a paragraph positioning the present findings against previously reported MSC-based therapies for ALD was missing from Section 3 (Discussion). A new paragraph has been added as the fourth paragraph of the Discussion and reads as follows:

“Our approach differs from previously reported MSC-based therapies for ALD in three respects. Clinically, autologous bone marrow-derived MSCs (BM-MSCs) delivered through the hepatic artery improved the Laennec fibrosis grade in 6 of 11 patients (54.5%) and the Child–Pugh score in 10 of 11 patients (90.9%) with alcoholic cirrhosis [30], and a subsequent multicenter phase 2 trial in 72 patients confirmed that autologous BM-MSC transplantation safely improved histologic fibrosis and liver function [31]. These benefits were nonetheless partial and variable, and both trials used unmodified cells that act only after reaching the liver. Preclinically, MSCs have been genetically enhanced with Bcl-2 [12], Smad7 [13], or HGF [14] to increase their efficacy in cirrhosis models, but these strategies have generally relied on viral transduction and deliver a single effector molecule. In contrast, the present study (i) uses a non-viral vector, thereby avoiding insertional mutagenesis and vector-directed immunity; (ii) drives a single upstream regulator, IFN-β, which mobilizes HGF and TRAIL together through the STAT pathway rather than one downstream effector; and (iii) acts primarily on the gut–liver axis, because the hepatoprotective effect observed here was attributable to HGF-mediated preservation of the intestinal barrier rather than to a direct action on hepatocytes. Preservation of the gut barrier is a mechanism that unmodified MSC therapy in earlier ALD studies did not address, and it may account for the additional benefit of ASC-IFN-β over ASC-Mock observed in this study.”

## Text Clarifications in the Abstract, Introduction, Results, Discussion and Materials and Methods

In the original publication [1], several passages have been revised for clarity and for completeness of reporting; none of these changes affects the data or the conclusions. In the Abstract, the opening sentences “Alcoholic liver disease (ALD) is a form of hepatic inflammation. ALD is mediated by gut leakiness.” now read “Alcoholic liver disease (ALD) is a form of hepatic inflammation that is mediated, in part, by gut leakiness.”; “adipose tissue-derived mesenchymal stem cells (ASCs)” is given in full at first mention; the comparator, given as “a control ASC”, is now identified as “control ASCs transfected with the empty vector alone (ASC-Mock)”; “blood endotoxin” has been corrected to “serum endotoxin”; and “mitigated the ethanol-induced down-regulation of cell death and permeability in Caco-2 cells” has been corrected to “attenuated ethanol-induced cell death and barrier disruption in Caco-2 cells”. In addition, minor editorial changes have been made throughout the article for grammar, consistency of terminology and completeness of first-mention definitions; these do not alter the meaning of any statement.

In Section 1 (Introduction), the sentence “In non-clinical studies, it has been found that alcohol-induced liver damage causes with intestinal damage, thereby inducing intestinal inflammation and leakiness.” now reads “In preclinical studies, alcohol-induced liver damage is accompanied by intestinal damage, which in turn induces intestinal inflammation and leakiness [5].”; the limitation of viral vectors, previously given as “However, ASCs engineered using viral-vector-mediated transduction are challenging to use clinically or obtain official approval as a drug due to the risk of viral vector-induced immune response.”, is now stated as “However, ASCs engineered by viral-vector-mediated transduction are difficult to translate clinically and to register as drug products, because viral vectors can provoke immune responses and carry a risk of insertional mutagenesis [15,16].” In the final paragraph of the Introduction, “HGF secreted by IFN-β-treated ASCs” has been corrected to “HGF secreted from IFN-β-overexpressing ASCs”, since the cells used were transfected to overexpress IFN-β rather than treated with it.

In Section 2 (Results), the following corrections have been made so that the text matches the data shown in the figures. In Section 2.1, “human IFN-β treatment (100 to 500 units/mL) induced human HGF expression” now reads “human IFN-β treatment (100 to 1000 units/mL) induced human HGF expression”, in accordance with Figure 1A,B. In Section 2.3, the description of the intestinal histology now reads “abnormal morphology (increased villus and crypt lengths) of the intestines was frequently observed in alcohol-exposed mice compared to sham (Figure 3A–D)”, in place of “abnormal shapes (increased villi length and decreased crypt length) of intestines were frequently observed in alcohol-exposed mice compared to sham”, in accordance with Figure 3C,D; and “plasma endotoxin” has been corrected to “serum endotoxin”, consistent with Section 4.7 and with the legend of Figure 3I. In Section 2.4, “Caco-2 cells co-treated with 40 mM ethanol and HGF (10 to 40 ng/mL) for 24 h showed reduced caspase 3 activity” now reads “Caco-2 cells co-treated with 40 mM ethanol and HGF (20 and 40 ng/mL) for 24 h showed reduced caspase 3 activity”, because the reduction was statistically significant only at 20 and 40 ng/mL (Figure 4C); “HGF treatment prevented ethanol-induced barrier permeability in Caco-2 cells” now reads “HGF treatment prevented ethanol-induced barrier disruption in Caco-2 cells, as assessed by transepithelial electrical resistance (TEER) and fluorescein isothiocyanate (FITC)-dextran flux”; and “Single treatment of IFN-β, TRAIL, or HGF does not affect caspase 3 activity and barrier permeability in control Caco-2 cells (Figure 4A–E)” now reads “Treatment with IFN-β, TRAIL, or HGF alone did not affect caspase 3 activity or barrier permeability in control Caco-2 cells (data not shown)”, because all panels of Figure 4 show ethanol-exposed cells and do not contain these single-treatment controls. In Section 5 (Conclusions), the typographical error “INF-β” has been corrected to “IFN-β”; the same error in Section 3 (Discussion) has also been corrected.

In Section 3 (Discussion), the following changes have been made. In the paragraph beginning “Our results demonstrated that intraperitoneal injection…”, the two closing sentences “IFN-β and TRAIL did not prevent ethanol-induced destruction of the epithelial barrier. However, chronic alcohol consumption may inhibit hepatic fibrosis and carcinogenesis in patients with liver cirrhosis or cancer.” have been replaced by “IFN-β and TRAIL alone did not prevent ethanol-induced disruption of the epithelial barrier in vitro. Nevertheless, their concurrent secretion from ASC-IFN-β may confer additional benefit in patients with alcoholic cirrhosis, in whom the risks of fibrosis progression and hepatocarcinogenesis are high.”, as the earlier second sentence did not convey the intended meaning. In the paragraph beginning “As lentiviral vectors usually carry…”, “we used a commercial non-viral vector to overexpress INF-β-mediated HGF” now reads “we used a commercial non-viral vector to drive IFN-β-mediated HGF secretion”, and the two closing sentences “ASCs themselves are home to the site of inflammation; hence, they can also induce transduction in other normal cells through viral vectors in normal tissues. Therefore, they have the advantage of countering safety concerns.” have been replaced by “In addition, ASCs home to sites of inflammation. When engineered with viral vectors, they may therefore transduce bystander cells in normal tissues, a risk that non-viral engineering avoids. Non-viral engineering thus offers a clear safety advantage.” In the paragraph beginning “A strategy for directly overexpressing HGF…”, the three closing sentences have been rewritten and now read “HGF overexpression increases the therapeutic efficacy of MSCs, but it cannot elicit the additional secretion of IFN-β or TRAIL. In contrast, overexpression of IFN-β in ASCs induces both TRAIL and HGF through the IFN-β/STAT pathway. Engineered ASCs that migrate to intestinal and hepatic tissue may therefore act synergistically, limiting local inflammation and promoting tissue repair through the combined action of these secreted factors.”, in place of the earlier wording, which referred to “a combination of the three cytokines secreted by the engineered ASC”. Finally, the concluding paragraph of the Discussion now begins “In summary, our study demonstrated…” instead of “In other words, our study demonstrated…”.

In Section 4 (Materials and Methods), the following corrections have been made. In Sections 4.1 and 4.2, the supplier location given for collagenase and for X-tremeGENE™ Transfection Reagents has been corrected from “Roche, Munich, France” to “Roche, Mannheim, Germany”. In Section 4.7 (Endotoxin Measurement), the handling of blood samples now reads “Blood was collected from the mice via cardiac puncture and allowed to clot at room temperature. After centrifugation (3000 rpm, 20 min, 4 °C), the serum was collected and frozen until use.”, replacing the earlier description of centrifugation “in a heparin tube (BD Biosciences, San Jose, CA, USA)”, which was inconsistent with the reference to serum in the same subsection and with the legend of Figure 3I. The heading of Section 4.9 has been corrected from “Serum Alanine and Aspartate Transaminase Measurements” to “Serum Alanine and Aspartate Aminotransferase Measurements”, and the corresponding terms in the text have been changed accordingly. In Section 4.10, the supplier of TRIZOL reagent, given as “Thermo Fisher Carlsbad, CA, USA”, is now given in full as “Thermo Fisher Scientific, Carlsbad, CA, USA”. In Section 4.5, “euthanized 1 h after the final binge alcohol injection” now reads “euthanized 1 h after the final binge alcohol administration”, the alcohol having been given orally. Apart from the description of blood handling in Section 4.7, no experimental procedure, measurement or result was changed. In Section 4.3, “FITC-D4” now reads “FITC-4k dextran”, matching the abbreviation used in the legend of Figure 4. In Section 4.5, “The mice were administered a single intraperitoneal injection of 2.0 × 106 ASCs/200 μL PBS on day 2 after receiving a second binge alcohol dose.” now reads “…immediately after the second binge alcohol dose.”, in accordance with the experimental design shown in Figure 2A and with Section 2.2. In Section 4.1, the typographical error “DMEM Sigma-(Aldrich, St. Louis, MO, USA)” has been corrected to “Dulbecco’s modified Eagle medium (DMEM; Sigma-Aldrich, St. Louis, MO, USA)”. In Section 4.2, “phosphate-buffered solution (PBS)” has been corrected to “phosphate-buffered saline (PBS)”.

## Standardization of the Control-Group Abbreviation

In the original publication [1], the cells transfected with the empty vector were referred to as “ASC-vec” in the Results text and as “ASC-Mock” in the legends of Figures 2 and 3. Throughout the article, this control group is now referred to consistently as “ASC-Mock”, defined at first mention as “ASCs transfected with the empty vector alone (ASC-Mock)”. The control group itself, and all data obtained from it, are unchanged.

## Figure Legends and Statistical Reporting

In the original publication [1], the legends of Figures 1–4 and Section 4.12 (Statistical Analysis) did not fully specify the unit of replication, the summary statistic and the statistical test applied. The legends of Figures 1–4 have been revised to state, for each panel, the number of replicate wells, Transwell inserts or mice per group (n) and the number of independent experiments; that the data are presented as the mean ± standard error of the mean (SEM); and that between-group comparisons were performed using the two-tailed Mann–Whitney U test. Accordingly, the description “Scatter plot. Horizontal bar; median.” in the legend of Figure 1 has been replaced. The underlying data and all reported *p* values are unchanged. The figures themselves are unchanged: all panels are bar graphs showing a group summary value with error bars, so the earlier description “Scatter plot. Horizontal bar; median.” did not correspond to the graphs shown. The median referred to in Sections 4.6 and 4.9 is the value used to summarize the repeated measurements obtained from each individual animal, and is distinct from the group-level statistic plotted in the figures, which is the mean ± SEM. The following corrections have also been made within the legends: in the legend of Figure 2, “Body weight was measured after the third injection of ethanol” now reads “Body weight measured after the third administration of ethanol”, the alcohol having been given orally; in the legends of Figures 2 and 3, the panels showing TNF-α, IL-1β and iNOS are now identified as “mRNA expression”; and in the legend of Figure 3, “(J) Median number of colonies.” now reads “(J) The number of Escherichia coli colony-forming units (CFUs) recovered from liver tissue.”

In addition, Section 4.12 (Statistical Analysis) has been revised and now reads as follows:

“All data are presented as the mean ± standard error of the mean (SEM). All in vitro experiments were performed with four replicates per treatment (*n* = 4 per group) and were repeated in three independent experiments. For the animal experiments, the number of mice analyzed per group is stated in the corresponding figure legend. Because the group sizes were small and normality could not be assumed, all between-group comparisons were performed using the two-tailed Mann–Whitney U test (GraphPad Prism 10.3.0; GraphPad Software, Boston, MA, USA). Statistical significance was set at *p* < 0.05.”

## Addition of the Abbreviations and Acknowledgments Sections

In the original publication [1], an Abbreviations list was not included. A list of the abbreviations used in the article has been added to the back matter. In addition, the following Acknowledgments section has been added:

“The authors used Claude (Anthropic, San Francisco, CA, USA) for English language editing during the preparation of this revised manuscript. The tool was not used to generate, analyze, or interpret any experimental data, nor to produce any figure. The authors reviewed and edited all text and take full responsibility for the content of the published article.”

The authors state that the scientific conclusions are unaffected. This correction was approved by the Academic Editor. The original publication has also been updated.

With this update, the Academic Editor is satisfied that the editorial process related to this article has been completed as per MDPI’s Editorial Process policy. The Editorial Office would like to thank the authors for their collaboration during this process.
